# Thymic stromal lymphopoietin is expressed in the intact central nervous system and upregulated in the myelin-degenerative central nervous system

**DOI:** 10.1002/glia.22662

**Published:** 2014-03-25

**Authors:** Maja Kitic, Isabella Wimmer, Milena Adzemovic, Nikolaus Kögl, Antonia Rudel, Hans Lassmann, Monika Bradl

**Affiliations:** Medical University Vienna, Center for Brain Research, Department of NeuroimmunologySpitalgasse 4, 1090, Vienna, Austria

**Keywords:** thymic stromal lymphopoietin, astrocytes, microglia, myelin degeneration

## Abstract

Thymic stromal lymphopoietin (TSLP) is an epithelial cytokine expressed at barrier surfaces of the skin, gut, nose, lung, and the maternal/fetal interphase. At these sites, it is important for the generation and maintenance of non-inflammatory, tissue-resident dendritic cell responses. We show here that TSLP is also expressed in the central nervous system (CNS) where it is produced by choroid plexus epithelial cells and astrocytes in the spinal cord. Under conditions of low-grade myelin degeneration, the numbers of TSLP-expressing astrocytes increase, and microglia express transcripts for the functional TSLP receptor dimer indicating that these cells are targets for TSLP in the myelin-degenerative CNS.

## Introduction

Epithelial cells at barrier surfaces like skin, gut, nose, and lung express the cytokine thymic stromal lymphopoietin (TSLP) (Kamekura et al., 2009; Reche et al., 2001). Changes in the tissue-specific expression of TSLP are associated with a number of diseases. For example, in patients with atopic dermatitis, asthma, or allergic rhinitis, TSLP expression is dramatically upregulated in the skin, lung, or nose (Kamekura et al., 2009; Ziegler and Artis, 2010), and is associated with type 2 inflammatory reactions (Ziegler and Artis, 2010). In patients with Crohn's disease, this molecule shows lower expression levels in colonic epithelial cells (Rimoldi et al., 2005), coinciding with the development of type 1 and type 17 inflammatory reactions (Ziegler and Artis, 2010). TSLP is a key molecule for the generation and maintenance of non-inflammatory, tissue-resident dendritic cell (DC) responses (Ziegler and Artis, 2010).

Since under pathological conditions, also microglial cells can differentiate to non-inflammatory, tissue-resident DCs (Fischer et al., 2000; Reichmann et al., 2002), we investigated whether TSLP is also expressed in the central nervous system (CNS), whether the expression of TSLP at this site changes under pathological conditions, and whether microglial cells express the appropriate receptors for TSLP.

## Materials and Methods

### Animals

For cell culture and isolation of microglial cells by FACSorting, proteolipid protein (PLP)-overexpressing transgenic and wildtype Lewis rats were obtained from the Decentral Facilities of the Institute for Biomedical Research, Medical University, Vienna. The genotype of the transgenic animals was determined by PCR analysis as described (Bradl et al., 1999).

For histology, archival CNS material from 10 to 12 months old PLP-transgenic Lewis rats and age-matched wildtype Lewis rats (Bradl et al., 2005) was used. In all these cases, the animals had been sacrificed by inhalation of an overdose of CO_2_, and perfused with 4% paraformaldehyde (PFA) prior to the dissection of brains and spinal cords. The tissue was post-fixed in 4% PFA for additional 18–24 hours and subsequently embedded in paraffin.

### Histological Analysis

For the analysis of TSLP expression in tissue slices, 2–4 µm thick sections were used without further pre-treatment (anti-mouse/rat TSLP; anti-Iba1), or after antigen retrieval for 1 hour in a commercial food steamer in 10 mM EDTA buffer pH 8.5 (anti-GFAP). The sections were rinsed in 0.05 M Tris-buffered saline (TBS) and incubated in Dako buffer (DakoCytomation, Vienna, Austria) containing 10% fetal calf serum (DB/FCS) for 20 min. The slides were incubated in blocking buffer at 4°C overnight with the following primary antibodies: polyclonal rabbit anti-mouse/rat TSLP (ab3, Sigma Aldrich; 1:50), polyclonal rabbit anti-Iba1 (Wako Chemicals GmbH, Neuss, Germany; 1:50), or mouse anti-rat glial fibrillary acidic protein (GFAP, Neomarkers, Fremont, CA; 1:200). The sections were then washed three times in TBS and incubated with biotinylated secondary antibodies (donkey anti-rabbit, 1:2,000, or sheep anti-mouse, 1:500; both antibodies from Jackson Immunoresearch) in DB/FCS for 1 hour at room temperature, followed by three times washing in TBS and incubation with avidin–peroxidase complex (1:100 in DB/FCS; Sigma) for 1 hour. After another washing step, labeling was visualized with 3,3′-diaminobenzidine-tetra-hydrochloride (DAB, Sigma) containing 0.01% hydrogen peroxide. All sections were counterstained with Meyer's hematoxylin, dehydrated, and mounted in Eukitt (Sigma).

### Astrocyte Cultures

These cultures were made essentially as described (Bradl et al., 2009; Hochmeister et al., 2008; Kivisakk et al., 2009; Sharma et al., 2010). Briefly, brains of 0–24 hours old rat pups were dissected, cleaned of meninges, homogenized in RPMI/10%FCS, and plated on poly-l-lysine-coated culture flasks. Confluent mixed glia cultures (after 7–8 days *in vitro*) were placed on a shaking platform to detach microglial and oligodendrocyte lineage cells (170 rpm, 37°C, overnight). The supernatant containing the detached cells was used for microglia cultures as described below. The adherent, astrocyte-enriched cells were detached by incubation with accutase (PAA Laboratories, Pasching, Austria; 5–10 min at 37°C), subsequently washed with RPMI/10% FCS and reacted with NMO-IgG [0.1 mg/mL, specific for surface epitopes of aquaporin 4 (AQP4) on astrocytes (Bradl et al., 2009)], control human IgG (Subcuvia, Baxter, Vienna; 0.1 mg/mL), or stain buffer alone (PBS/0.2% BSA) for 30 min at 4°C. After washing, the secondary antibodies were applied (donkey anti-human-Cy3 (Jackson ImmunoResearch; 1:100) or biotinylated sheep anti-human (GE Healthcare/Amersham, Vienna, Austria; 1:100), followed by an incubation with streptavidin-Cy2 (Jackson ImmunoResearch; 1:75). Pure AQP4^+^ astrocytes were obtained by FACSorting, using a FACSAria sorter with a 70-μm nozzle and DiVa software, or the FACScalibur system using the Cellquest Pro-software (all Becton Dickinson, San Jose, CA).

### Choroid Plexus Epithelial Cultures

Adult Lewis rats were sacrificed by an overdose of CO_2_, their brains dissected and sagitally sliced, and the choroid plexuses excised from the two lateral and from the fourth ventricle. This tissue was first incubated with 1 mg/mL collagenase in PBS (20 min, 37°C) and centrifuged (800*g*, 5 min). Afterward, the cell pellet was further subjected to five rounds of brief trypsin/DNase (0.025% Trypsin–EDTA and 12.5 μg/mL DNase I in PBS) treatment, followed by centrifugation (800*g*, 5 min). Finally, the cells were resuspended in Ham's F-12/DMEM (1:1) supplemented with 10% fetal bovine serum (FBS), 2 mM l-glutamine, 100 U/mL penicillin/streptomycin, 5 μg/mL insulin, 5 μg/mL transferrin, 5 ng/mL sodium selenite, 10 ng/mL EGF, 2 μg/mL hydrocortisone, 5 ng/mL basic fibroblast growth factor, 500 µM hypoxanthine, and 250 µg/mL hydroxy-l-proline, and plated on 35 mm culture dishes coated first with 0.01% collagen and then with 10 μg/mL laminin. Cells were cultured further in the presence of 20 µM cytarabin for the first 3 days *in vitro*. Purity of cultures, as estimated by an immunofluorescent staining of aquaporin-1 (AQP-1, marker of choroidal epithelial cells), morphology, and expression of transthyretin (Ttr) was approximately 98%.

### Microglia Isolation and Characterization

#### Isolation

About 5- to 12-month-old PLP-transgenic and wildtype Lewis rats were sacrificed with an overdose of CO_2_, transcardially perfused with PBS, and their spinal cords were carefully dissected, stripped from meninges, and passed through cell strainers. Microglia cells and macrophages were then purified from this suspension using Percoll (density 1.0489 g/mL) gradient centrifugation (Nycomed-Pharma AS, Oslo, Norway). Afterward, a cell surface staining of the cells was made, using FITC-labeled anti-CD45 (recognizing leukocyte common antigen, from AbD Serotec, Düsseldorf, Germany). Since microglia cells express substantially less cell surface CD45 than macrophages (Ford et al., 1995), CD45^low^ microglia cells could be clearly distinguished from CD45^high^ macrophages, and therefore be isolated by FACSorting, using a FACSAria sorter with a 70-μm nozzle and DiVa software, or the FACScalibur system using the Cellquest Pro-software (all Becton Dickinson, San Jose, CA).

#### Characterization by flow cytometry

Microglial cells from wildtype and PLP-transgenic rats were simultaneously stained with FITC-labeled anti-CD45 and biotinylated anti-CD80 (B7.1), anti-CD86 (B7.2), and isotype control antibodies (all from AbD Serotec). The biotinylated antibodies were finally detected using APC-conjugated anti-biotin antibodies (Miltenyi Biotec, Bergisch Gladbach, Germany). For flow cytometry, gates were set on CD45^low^ cells, and the expression profile of the gated cells was analyzed using the FACScalibur system and Cellquest Pro-software.

#### Gene expression studies

Microglial cells of seven wildtype and eight PLP-transgenic rats were used. The RNA of these cells was isolated as described above and sent to ImaGenes (Berlin, Germany) for hybridization to Agilent Multiplex 4×44K Rattus Norvegicus Arrays, for scanning and quantile normalization. The gene IDs of the sequences studied (Cd40, Cd68, Cd80, Cd86, Ox40L, CCL17, CCL22, and RT1-B^b^), their accession numbers in the GenBank sequence database, and the sequence of the nucleotide probes used to identify these genes are summarized in Table1.

**Table 1:** Sequence of the Nucleotide Probes Used for Gene Expression Studies

Gene	Accession number	Probe sequence (5′-> 3′)
Cd68	NM_001031638	ATCCTCATTTCTTCAGCATGCAATTGACTCAACAGAGTTATCTCCCTTCTCTGTCTTTAAA
RT1-B^b^	NM_001004084	CAGCTGTGACAGTTGTGAAATACCCTAGCTTCTGATAACAGAATGAGTTACTTCTTCCCAA
Cd86	NM_020081	TAAGCAAGGATACCCGAAACCTACAAAGATGTATTTTTTGATAACTAATA
Cd80	NM_012926	TGAATCTGAGCTGTACACCATTAGTAGCCAACTAGACTTCAACACGACGTACGACCACTTA
Ccl17	NM_057151	CTGCACAGACCCCAAAGACAAGCATGTGAAGAAGGCCATCAGACACCTGAAAAACCAAAGA
Ccl22	NM_057203	GATATCTGTGCTGACCCCAGGATGCTCTGGGTGAAGAAGATACTCCACAAGTTGGCCTAGA
Ccl22	NM_057203	AAATAAATTTGCTTGCTCCTTTGGAGGGAACAGTGGCCTGGCTTAGCTGAGTGAATGGATA
II12a	NM_053390	ACTTCAGAGCCACAATCATCAGCAGATCACTCTGGACAGAAACATGCTGATGGCTATTGAA
II12b	NM_022611	TTGGTCCACCGAGATTTTAAAAAATTTCAAAAATAAGACTTTCCTGAAGTGTGAAGCACCA
Cd40	NM_134360	ATGGAGGAAAAGCTTTGGCGTCAGGGGTCCGCAGTAATATCTACAGAGTGCAGCAATGCAA
Cd40	NM_134360	ACTGCACAGCTCTTGAGAAGACCCAATGCCAACCGTGCGACTCAGGCGAATTCTCAGCTCA
Ox40L	NM_053552	TTGTGTTACAATACAGTGCGTATGTCTTGAACCTCCAGAAAGTCTGAAGGCTACTAATCCA

### Isolation of Meningeal Antigen Presenting Cells

Meningeal antigen presenting cells were isolated according to modified protocols (Hochmeister et al., 2008; Kivisakk et al., 2009). Briefly, rats were perfused with 50 mL PBS and meninges were carefully dissected from the brain and spinal cord tissue. This was followed by a 1 hour incubation in ice-cold HBSS (without Ca^2+^ and Mg^2+^; BioWhittaker) supplemented with 2 mM EDTA (Serva) and 5 mM HEPES (Sigma). Cells were further mechanically dissociated from meningeal tissue by passage through a 70-µm cell strainer. After centrifugation (300*g*, 1 min/mL), cells were either re-suspended in stain buffer (PBS/0.2% BSA) alone, or in stain buffer containing mouse anti-rat MHC class II RT1B antibody (OX-6 clone, AbD Serotec, 1:100) or IgG1 isotype control (Dako Cytomation, 1:100) and incubated for 30 min at 4°C. Cells were then washed three times with stain buffer and incubated for another 30 min with DyLight 549—conjugated donkey anti mouse antibody (Jackson ImmunoResearch, 1:100). The MHC class II+ cells were isolated by FACSorting as described above, and immediately afterward used for RNA isolation and PCR analyses.

### Rat Brain Endothelial Cultures

These cultures were established and characterized as recently published (Kitic et al., 2013).

### Characterization of Cell Cultures

#### RNA isolation, cDNA synthesis, and polymerase chain reactions

Cellular RNA was isolated using the RNeasy Mini Kit (Qiagen, Vienna, Austria) according to the manufacturer's instructions. To avoid contamination of the RNA sample with genomic DNA, the following procedures were carried out: 50 µL RNA sample was mixed with 7 µL 10× Dnase buffer, 2 µL RiboLock RNase inhibitor, 7 µL DNase 1 (all from Fermentas), and 4 µL RNase-free water (GIBCO, Invitrogen) and incubated for 30 min at 37°C, then 7 µL 25 mM EDTA (Fermentas) were added, and the samples were further incubated for 10 min at 65°C. After RNA clean-up, the RNA was eluted in 30 µL RNase-free water and subsequently annealed with a T7-N7 primer (5′–CCAAGCTTCTAATACGACTCACTATAGGGAGANNNNNNN-3′; incubation at 70°C for 5 min), supplemented with 10 µL 5× M-MLV RT Buffer (Promega), 3 µL dTNPs (Roche), 3 µL RiboLock RNase I Inhibitor (Fermentas), and 5 µL RNase/DNase-free H_2_O (GIBCO, Invitrogen), and incubated at 40°C for 2 min. Reverse-transcriptase [M-MLV Reverse Transcriptase, RNaseH, point mutant, 200 U/µL (Promega)] was added to each sample. The samples were incubated first at RT for 10 min, then at 40°C for 50 min, and finally at 70°C for 15 min.

Quantitative real-time polymerase chain reaction (qPCR) was performed using the QuantiFast™ SYBR® Green PCR Kit (Qiagen) in a StepOnePlus RT-PCR system (Applied Biosystems), according to the manufacturer's instructions. The following QuantiTect Primer Assays (Qiagen) were used for gene amplification: Rn_Crfl2_1_SG for Crlf2 (Tslpr), Rn_Gapdh_1_SG for Gapdh, Rn_Gfap_1_SG for Gfap, Rn_Il7r_2_SG for Il7r, and Rn_Ttr_1_SG for Ttr. For the detection of Tslp, transcript self-designed primers (Tspl-forward: 5′-TCCTGAAAATCGACCACCAT-3′ and Tslp-reverse: 5′-GATTGTGGCTTTCCTGCATT-3′) were used, with a final concentration of 0.5 μM for each primer. The following cycling conditions were applied: initial heat activation (5 min, 95°C), followed by 40 cycles of denaturation (10 s, 95°C), and annealing/extension (30 s, 60°C). The absence of non-specific amplification was determined by a melt curve analysis. All reactions were run in duplicates or triplicates. Normalized gene expression levels were calculated using the equation: 2^−ΔCt^ = 2^−[Ct(GOI)−Ct(HKG)]^ (*GOI*, Gene of interest; *HKG*, house-keeping gene).

Further PCR analyses were done using the FastStart Taq Polymerase kit (Roche Applied Science, Vienna, Austria). One reaction consisted of: 5 µL 10× PCR buffer (200 mM Tris–HCl, pH 8.4, 20 mM MgCl_2_), 1 µL 10 mM dNTP mix, 1 µL forward primer (100 pmol/µL), 1 µL reverse primer (100 pmol/µL), 0.4 µL polymerase (5 U/µL), 1 µL cDNA, and 40.6 µL H_2_O. The following primer pairs were used: Gfap (forward: 5′-TTGTTTGCTAGGCCCAATTC-3′; reverse: 5′-CCTCGGGATCTTTTCCTTTC-3′; product size: 356 bp); GAPDH (forward: 5′-GGCATTGCTCTCAATGACACC-3′; reverse: 5′-TGAGGGTGCAGCGAACTTTAT-3′; product size: 311 bp); TSLP (forward: 5′-TCCTGAAAATCGACCACCAT-3′; reverse: 5′-AATGCAGGAAAGCCACAATC-3′; product size: 215 bp); Transthyretin (TTR; forward: 5′-GGCTCACCACAGATGAGAAGTTC-3′; reverse: 5′-ACAAATGGGAGCTACTGCTTTGGC-3′; product size: 269 bp); TSLPR (forward: 5′-TTTCTGTTGGACAGCGTCAG-3′; reverse: 5′-TGCTGCACCTCATATTCCAG-3′; product size: 230 bp), and IL-7Rα (forward: 5′-GCAACTGTACACGGTGCAAACTGG-3′; reverse: 5′-TCTGGAGCTTGGCAGCAAGTCT-3′; product size: 204 bp). The reaction mixture was subjected to an initial denaturation step (10 min, 95°C), and then to 30, 35, and 40 cycles of denaturation (30 s, 95°C), annealing (30 s; 50°C for TSLP, 51°C for GFAP, 53°C for GAPDH, 55°C for TSLPR, and IL-7Rα, 57°C for TTR), and elongation (30 s, 72°C). The reaction was terminated with final extension for 10 min at 72°C, and PCR products were detected by agarose gel electrophoresis. Sequencing of the PCR products (VBC Biotech, Vienna, Austria) confirmed their identity.

#### Statistical analysis

Statistical analyses were conducted using Kruskal–Wallis test, followed by pair-wise Mann–Whitney *U*-tests and Bonferroni correction. The *P*-values are results of two-sided tests. Data are represented in boxplots that show the range of numbers of TSLP^+^ cells (whiskers), with 50% scores (interquartile range, boxes) centered around median values (horizontal lines within boxes). Analyses were performed using PASW Statistics 18 software (SPSS Inc.).

## Results

### TSLP Expression and Recognition in the Intact CNS

**Figure 1:**
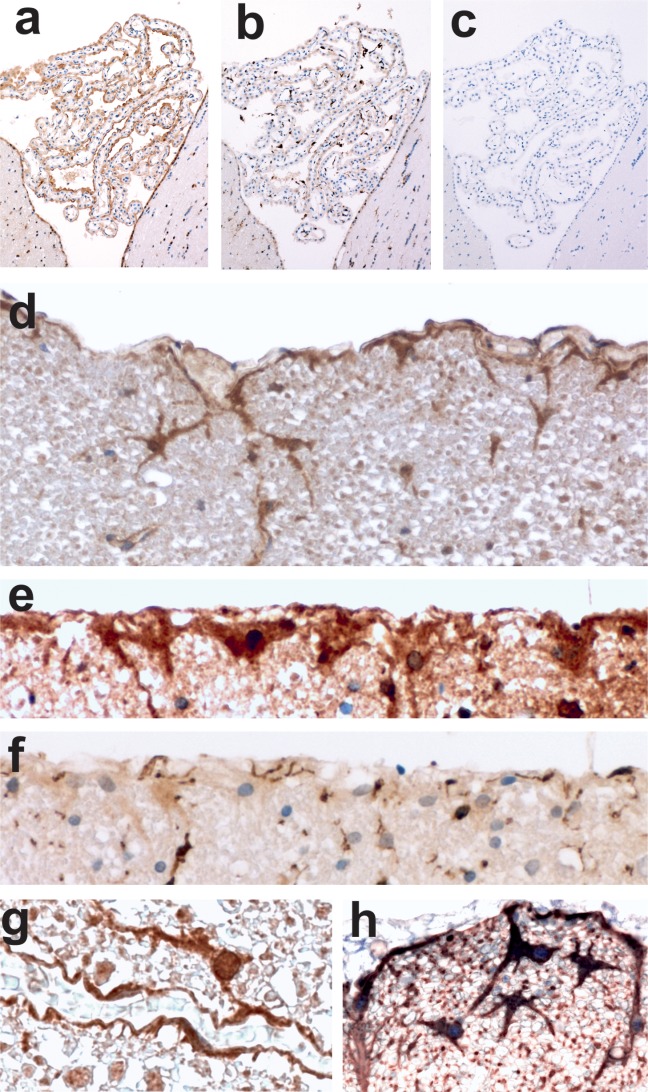
Expression of TSLP in the central nervous system of Lewis rats. Shown here are choroid plexus (a–c) and spinal cord (d–h). Consecutive sections of choroid plexus were reacted with anti-TSLP (a, brown reaction product), anti-Iba-1 (b, brown reaction product), or without first antibody (c), and were counterstained with hematoxylin to reveal nuclei in *blue* color. In the spinal cord, TSLP is expressed at the subpial (d, e, h) and perivascular (g) glia limitans. For further control, consecutive sections of the spinal cord were reacted with anti-TSLP to reveal staining of astrocytes (e), and with anti-Iba-1 to reveal the absence of astrocytic and the presence of microglial staining specific for this antibody (f). Double staining of sections with anti-TSLP (*blue*) and anti-GFAP (*brown*) antibodies confirmed TSLP expression by astrocytes (h).

In the spinal cord of healthy Lewis rats, GFAP^+^ astrocytes at the superficial glia limitans displayed cytoplasmatic and often also nuclear reactivity for TSLP. The numbers of TSLP^+^ astrocytes were highly variable, independent of the location of the spinal cord sections along the neuraxis (Fig. 1). Weaker staining was seen in astrocytes and astrocytic foot processes at the perivascular glia limitans of the white matter (Fig. 1). Throughout spinal cord gray matter, diffuse TSLP reactivity was noted, and only few cells showed TSLP expression levels above this background reactivity.

In the brain, TSLP-reactive astrocytes were essentially absent from the perivascular or subpial glia limitans (data not shown). Instead, choroid plexus cells were positive for TSLP (Fig. 1). We also detected nuclear and cytoplasmatic TSLP expression in some brain neurons. The most prominent reactivity was found in neurons located in the substantia nigra, lemniscus medialis, nucleus ruber, and decussatio tegmenti ventralis, while TSLP expression was essentially absent in neurons located within the adjacent interpeduncular nucleus. Also hippocampal neurons, Purkinje cells, and other neuronal cells throughout the brain parenchyma showed nuclear staining for TSLP (Supp. Info. Fig. 1). This finding was surprising, since neither TSLP knock-out animals (Reardon et al., 2011) nor TSLP-overexpressing transgenic mice (Osborn et al., 2004) display any neurological abnormalities. We did not further analyze neuronal TSLP expression in more detail.

**Figure 2:**
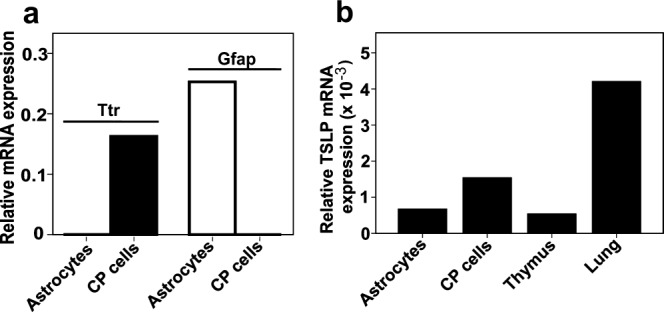
Confirmation of the expression of TSLP in astrocytes and choroid plexus epithelium. (a) The purity of the cultures used in this study was determined by real-time PCR, and was documented as mRNA expression of the choroid plexus (CP)-specific marker transthyretin (Ttr) and the astrocytic marker glial fibrillary acidic protein (Gfap) in relation to the house keeping gene glyceraldehyde-3-phosphate-dehydrogenase (Gapdh). (b) Real-time PCR to determine TSLP expression in relation to Gapdh, using cultured astrocytes and choroid plexus cells, and thymus and lung tissue. TSLP expression in astrocytes and choroid plexus epithelial cells was further confirmed by endpoint—polymerase chain reactions (data not shown). Please note that we were unable to identify organs without any TSLP expression as negative control.

To further confirm the expression of TSLP in astrocytes and choroid plexus epithelium, we established cultures of these cells and searched for the presence of TSLP transcripts by qPCR. TSLP mRNA was detectable in both types of cells indicating that they are able to produce TSLP (Fig. 2).

**Figure 3:**
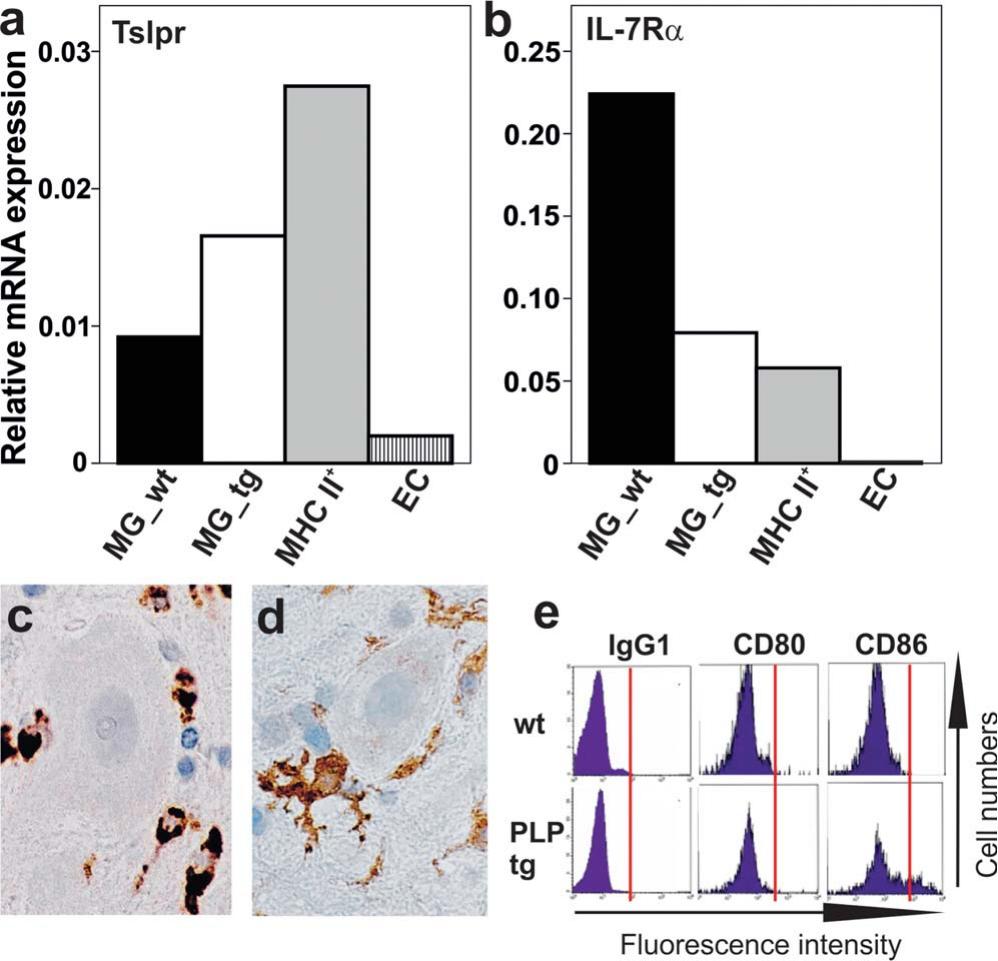
The expression of TSLP receptor chains in microglia and in MHC class II+ cells of the meningeal DC network. (a, b) FACSorted microglia of 6 wildtype (MG_wt) and 6 PLP-transgenic (MG_tg) 10–12 months old Lewis rats, FACSorted MHC class II+ cells of the meningeal DC network isolated from 13 wildtype 5 to 6 month old Lewis rats, and purified rat brain endothelial cells (EC) were tested by qPCR for the expression of both chains of a functional TSLP receptor, that is, for TSLPR (a) and IL-7Rα (b) in relation to the house keeping gene glyceraldehyde-3-phosphate dehydrogenase (GAPDH). The expression of Tslpr was further corroborated by end-point polymerase chain reactions, and the expression of the IL-7Rα by gene expression studies (data not shown). Microglia in the spinal cord gray matter of PLP-transgenic rats was stained with ED1 (c) to reveal the presence of CD68, and with OX-6 (d) to reveal the presence of MHC class II products. In both cases, positive reaction products are *brown*, and the nuclei counterstained with hematoxylin are *blue*. (e) Flow cytometric analysis of microglia isolated from the wt or PLP-transgenic CNS, using IgG1 as an isotype control, and antibodies against CD80 and CD86. Note the upregulation of CD86 on microglia derived from PLP-transgenic rats.

It has been described that TSLP preferentially stimulates myeloid cells (Qiao et al., 2011; Reche et al., 2001), and that DCs and monocytes coexpress TSLPR and IL-7Rα (Reche et al., 2001). Since microglia and monocytes share the ability to differentiate along the macrophage and DC lineage under several different pathological conditions (Fischer and Reichmann, 2001; Reichmann et al., 2002; Santambrogio et al., 2001), we analyzed by qPCR whether DCs from the meningeal DC network or microglial cells from the spinal cord contain the molecular machinery needed for TSLP recognition, that is, a functional TSLP receptor dimer consisting of the TSLP-specific receptor chain (TSLPR) and the common IL-7 receptor alpha chain (IL-7Rα). We found that the meningeal MHC class II^+^ DCs and spinal cord microglia produced transcripts encoding both the IL-7Rα chain and the TSLPR and were hence able to form a functional receptor for TSLP recognition (Fig. 3).

### TSLP Expression and Recognition is Altered in the Myelin-Degenerative CNS

**Figure 4:**
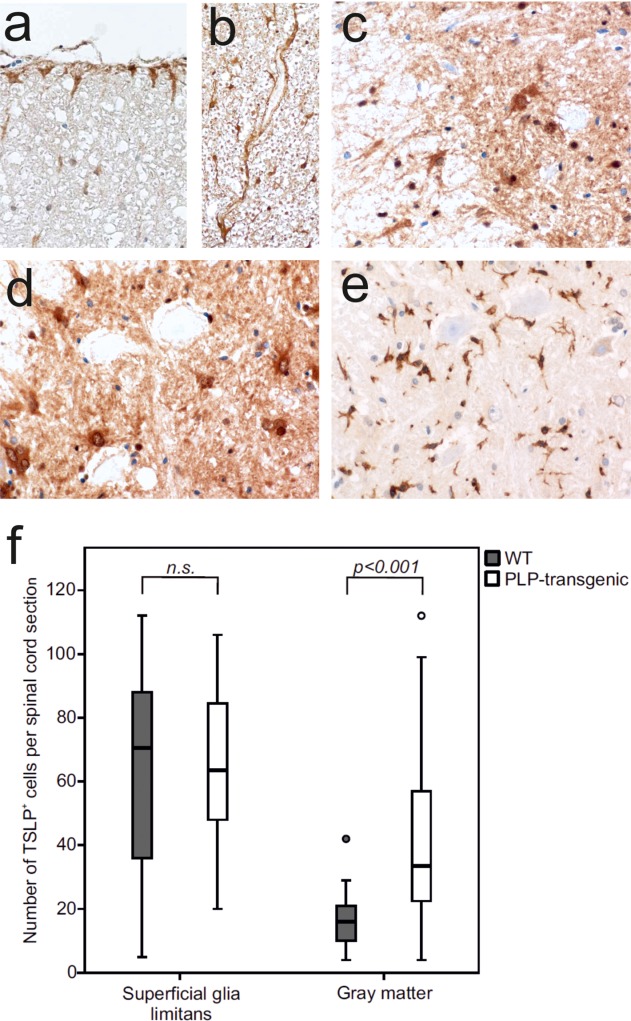
TSLP expression in the myelin-degenerative spinal cord of PLP-transgenic Lewis rats. All tissue samples derived from aged PLP-transgenic Lewis rats with chronic, subclinical myelin degeneration, which is especially pronounced in CNS gray matter (Bauer et al., 2002; Bradl et al., 1999). The spinal cord sections were reacted with anti-TSLP antibodies (a–d) or anti-Iba1 (e) (brown reaction products) and counterstained with hematoxylin to visualize nuclei (blue). Shown here are TSLP^+^ astrocytes at the subpial (a) and perivascular (b) glia limitans, in gray matter (c,d), and in white matter close to the gray/white matter junction (c). The rabbit anti-Iba1 antibody used as control does react with microglia, but not with neurons or astrocytes (e). (f) The number of TSLP^+^ cells in spinal cord gray matter (thoracic and lumbar segments) differs between wildtype Lewis (*gray bars*) and PLP-transgenic rats (*open bars*) according to Mann–Whitney *U*-tests (*P* < 0.001). In the superficial glia limitans, no significant difference in cell number was observed. Three thoracic and three lumbar spinal cord sections were analyzed per animal, using six PLP-transgenic and 5 wt Lewis rats.

We then analyzed the astrocytic TSLP expression in the myelin-degenerative spinal cords of PLP-transgenic Lewis rats. These animals are characterized by low-grade subclinical myelin degeneration and attempts of myelin repair, astrogliosis, and activation of microglia (Bradl et al., 1999). At the superficial glia limitans of lumbar and thoracic spinal cord of PLP-transgenic animals, the numbers of TSLP-reactive astrocytes were similar to those observed in site-matched wildtype spinal cords (Fig. 4). However, in PLP-transgenic animals, significantly more gray matter astrocytes in lumbar and thoracic spinal cord were TSLP-positive (Fig. 4). The location of the TSLP-reactive astrocytes closely reflected the pathological changes of PLP-transgenic rats, which were more pronounced in spinal cord gray than in white matter (Bauer et al., 2002). We then isolated microglia from the CNS of PLP-transgenic Lewis rats, and analyzed these cells by qPCR. We found transcripts for IL-7Rα, and low amounts of transcripts encoding TSLPR (Fig. 3). This finding clearly indicated that microglia in the myelin-degenerative CNS can produce TSLP receptor dimers.

### Microglial Properties in the Myelin-Degenerative CNS

**Figure 5:**
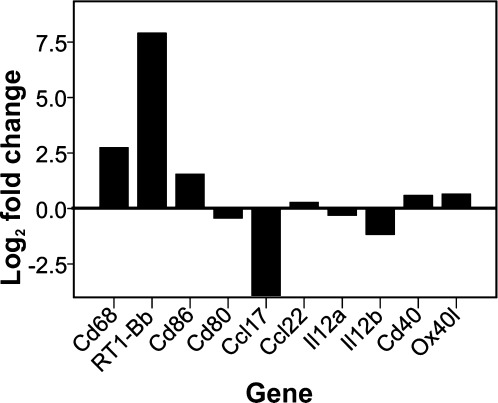
The phenotype of microglia isolated from PLP-transgenic rats. Shown here are log2-fold changes in the expression of TSLP-instructed transcripts in microglia isolated from PLP-transgenic animals, as compared with their wildtype derived counterparts. Note the upregulation of the microglial activation marker CD68, of MHC class II molecules (as an example, RT1-B^b^ is shown), of the costimulatory molecule CD86, of CD40 and Ox40 ligand, and the clear downregulation of IL-12b and of CCL17 in PLP-transgenic microglia in comparison to its wildtype counterpart. The microglial cells tested derived from seven wildtype and eight PLP-transgenic rats.

We could not detect the expression of functional TSLP receptor dimers by immunohistochemistry, probably due to expression levels below the limit of detection. Therefore, we used a different approach to study the effect of TSLP on microglia in the myelin-degenerative CNS. We isolated microglia from the spinal cords of PLP-transgenic rats and their wildtype Lewis counterparts and studied the expression of genes known to be affected by TSLP, that is, MHC class II products, the costimulatory molecules CD40, CD80 and CD86, OX40L, CCL17, and CCL22 (Zhou et al., 2005). Compared with their wildtype Lewis rat-derived counterparts, microglia isolated from the spinal cord of PLP-transgenic rats upregulated MHC class II products, showed enhanced expression of the costimulatory molecules CD40 and CD86 but not of CD80, and transcribed more OX40L products. mRNA coding for CCL22 and CCL17 was either unchanged or downregulated, respectively (Fig. 5). Hence, microglial cells revealed some, but not all features of TSLP-instructed cells.

## Discussion

TSLP is an epithelial cell-derived cytokine that is typically expressed at barrier surfaces of the body, that is, in the skin, gut, nose, lung, and at the maternal–fetal interphase, and plays an important role in the crosstalk between epithelial cells and DCs needed for the induction of physiological or pathological immune responses (Bogiatzi et al., 2007). In light of the epithelial expression pattern of TSLP it is not unexpected that also choroid plexus epithelial cells produce TSLP, possibly for the communication with DCs found under physiological conditions in the choroid plexus (McMenamin, 1999; McMenamin et al., 2003). However, it is much more surprising that astrocytes can produce TSLP as well, both in the intact and in the diseased spinal cord, and that TSLP is located both in the cytoplasm and in the nucleus.

The presence in the nucleus is not a unique feature of TSLP, but has also been noted for other cytokines like high mobility group box 1 (HMGB1), interferon-γ, interleukin-16, and interleukin-1 family cytokines (for review see Luheshi et al., 2009). Interestingly, in the IL-1 system, nuclear location was most often described for pro-IL-1α, and was associated with proliferation, induction of gene expression and migration (Luheshi et al., 2009). It is tempting to speculate that cytoplasmic/secreted and nuclear TSLP show a similar dual functionality.

TSLP reactivity of astrocytes is highly variable. This may reflect astrocyte heterogeneity (Black et al., 1993; Pfrieger and Slezak, 2012) whose effects on chemokine and cytokine production have already been described (Morga et al., 1999, Fitting et al., 2010).

In the intact CNS, TSLP expression by choroid plexus epithelial cells in the brain, and by astrocytes at the glia limitans in the spinal cord might regulate the survival and the effector functions of TSLP receptor dimer-expressing MHC class II^+^ cells in the meningeal/perivascular DC network.

However, in the myelin-degenerative spinal cords of PLP-transgenic Lewis rats, both the extent of TSLP expression and the cellular targets of TSLP change. Then, additional astrocytes become TSLP-positive. Interestingly, these astrocytes were predominantly located in the spinal cord gray matter, a compartment characterized by low-grade myelin degeneration, oligodendrocyte stress/death and microglia activation (Aboul-Enein et al., 2004; Bradl et al., 2005; Grundtner et al., 2007). In the same animals, microglial cells in the spinal cord expressed transcripts for both subunits of the TSLP receptor, suggesting that these cells are potential TSLP targets. TSLP might play a role in the activation and proliferation of these microglial cells, since it induces the phosphorylation and activation of signal transducers and activators of transcription (STATs), most prominently of STAT5 (Roan et al., 2012), which serves as a molecular regulator of cell fate, influencing proliferation, differentiation and apoptosis (Nosaka et al., 1999). Moreover, TSLP targets the same signal transduction pathway as granulocyte/monocyte-colony stimulating factor (GM-CSF) does (Liva et al., 1999; Yamaoka et al., 1998), a molecule involved in the control of microglia proliferation (Liva et al., 1999), activation (Natarajan et al., 2004) and initiation of microglia differentiation along the DC lineage (Hochmeister et al., 2008; Santambrogio et al., 2001). It is even possible, that TSLP and GM-CSF jointly act in the PLP-transgenic CNS, since microglia of PLP-transgenic rats also approximately two-fold upregulate the expression of Csf2rb1 (the GM-CSF/IL-3/IL-5 receptor common beta subunit; data not shown).

Microglial cells of PLP-transgenic rats do not show all features of TSLP-instructed DCs, since they upregulate transcripts for MHC class II products, CD40, CD86, and OX40L, but not for the chemokines CCL17 or CCL22. The most likely explanation for this finding is the fact that microglial cells *in vivo* can only partially differentiate along the DC lineage *in vivo*, and are arrested at an immature stage (Fischer et al., 2000; Fischer and Reichmann, 2001; Reichmann et al., 2002).
